# Thyroid cancer among patients with thyroid nodules in Yemen: a three-year retrospective study in a tertiary center and a specialty clinic

**DOI:** 10.1186/s13044-020-00082-x

**Published:** 2020-06-06

**Authors:** Butheinah A. Al-Sharafi, Jamila A. AlSanabani, Ibraheem M. Alboany, Amani M. Shamsher

**Affiliations:** 1 Department of Internal Medicine, Sana’a University, Sana’a, Yemen; 2 Department of Surgery, Sana’a University, Sana’a, Yemen; 3 Department of Radiology, University of Science and Technology, Sana’a, Yemen; 4 Department of Pathology, University of Science and Technology Hospital, Sana’a, Yemen

**Keywords:** Thyroid nodules, Bethesda, Thyroid cancer, Yemen, Thyroid surgery, Risk of malignancy, Fine-needle aspiration

## Abstract

**Background:**

The prevalence of thyroid cancer is increasing worldwide. No previous data are available on the prevalence of thyroid cancer in Yemen. We performed this study to determine the prevalence of thyroid cancer among patients with thyroid nodules in Yemen.

**Methods:**

A retrospective chart review was performed for 550 patients with thyroid nodules who underwent fine needle aspiration and/or thyroid surgery at a private endocrine clinic and at an endocrine clinic in a tertiary hospital in Yemen over a 3 -year period from October 2016–2019. The prevalence of thyroid cancer; the sonographic findings, Bethesda classification, age, sex, thyroid stimulating hormone (TSH) levels of the patients; and the nodule size and number were reviewed.

**Results:**

A total of 550 charts were reviewed [501 females (91.1%) and 49 males (8.9%)]. The thyroid cancer prevalence among the patients was 13.8% (CI = 10.9–16.7), and the mean age of the patients was 38.5 years (SD = 12.2). The TSH level and the rate of cancer were significantly related (P = 0.01), but no significant difference in the prevalence of thyroid cancer was found between females (13.4%) and males (18.4%) (P = 0.334). When correlating the rate of cancer with the ultrasound guided fine needle aspiration (UG-FNA) result, those with Bethesda system category III and IV, V and VI had malignancy rates of 20.8, 27.2, 52.4 and 69.2%, respectively. Thyroid nodules highly suspicious for malignancy on ultrasound had a 70% cancer diagnosis rate. The most common thyroid cancer was papillary cancer (71%), followed by follicular cancer (23.7%). Among those undergoing surgery, 44.2% had thyroid cancer, and 5.2% had a premalignant diagnosis.

**Conclusion:**

Thyroid cancer has a higher prevalence in Yemen than in other middle eastern countries. Our study also reports a higher rate of follicular thyroid cancer than that in other published data, which has to be confirmed by further studies. The malignancy and premalignant diagnosis rate was ~ 50% in our patients who underwent surgery. Many centers in Yemen still do not perform FNA before thyroid surgery. It is important that other centers in the country start emphasizing the need for FNA before surgery. This will decrease the number of unnecessary surgeries and associated complications.

## Introduction

Thyroid cancer is the most common form of endocrine gland cancer, accounting for 1% of all human neoplasias. It is estimated to have an annual incidence of 0.5–10/100000 people worldwide [1]. Thyroid nodules are palpable in 5% of the population on examination of the thyroid and detected in 50–67% of those undergoing thyroid ultrasound [2, 3]. The incidence of cancer differs from country to country; thyroid cancer occurs in approximately 5–15% of patients with thyroid nodules [4, 5], with the highest rate recorded in Korea (62.5/100,000). Other countries with high incidence rates include the USA (13.2), Canada (12.7), Turkey (10.9) and Italy (10.8) [6, 7]. Other studies from the Middle East showed thyroid cancer in 10.5% of patients with thyroid nodules in Jordan [8] and rates of 5% in Saudi Arabia in one study [9] and 10.4% in another [10]. In the United Arab Emirates, the prevalence was 9% among those of Emirati origin [11] and 5% among those with thyroid nodules in Pakistan [12]. Papillary thyroid cancer is the most common type of thyroid cancer, accounting for 80–95% of all thyroid cancers, with an incidence rate that has continued to increase globally in recent decades [13]. In this study, we retrospectively reviewed the charts of all patients who underwent UG-FNA and/or thyroid surgery during a 3-year period in a specialized endocrine clinic and in an endocrine clinic at a tertiary hospital in Sana’a, Yemen. Our goal was to determine the rate of thyroid cancer among our patients, the rate of cancer among the different Bethesda system categories and the correlation with thyroid nodule appearance on ultrasound. We also assessed the rate of cancer according to sex, age, number of nodules, TSH levels and size of the nodules.

## Methods

### Study design and settings

This is a retrospective analysis of a cohort of Yemeni patients [501 females (91.1%) and 49 males (8.9%)] with thyroid nodules who underwent ultrasound-guided FNA and/or surgery from October 2016 to October 2019 at an endocrinology private clinic or seen by the same endocrinologist at the University of Science and Technology Hospital (USTH) in Sana’a, Yemen (the ages of the patients ranged from 10 to 87 years old). The majority of the patients were seen at the specialty clinic 80.2% (N = 441), 15.6% (N = 86) were seen in the hospital, and a small number of patients [4.2% (N = 23)] were seen in other centers and then referred. All ultrasounds and UG-FNAs in the clinic were performed by a single radiologist, and those in the hospital were performed by one of the specialists in the radiology department. TSH was assessed in all patients, and patients were included in the study regardless of their thyroid state (euthyroid, hypothyroid or hyperthyroid).

### Chart review

The characteristics of all patients who underwent UG-FNA were obtained from their medical records. The age at the time of UG-FNA, sex, and TSH level were recorded. The age was grouped as < 20 years, 21–30, 31–40, 41–50, 51–60 and >  60. TSH was grouped as < 0.4 mU/L, 0.4–0.9, 1–1.7, 1.8–4.5, or >  4.5. Medications were grouped into no thyroid medications, L-thyroxine and antithyroid medications. Nodules were either single or multiple, and for the nodule size, the largest dimension of the largest nodule was noted. The ultrasound findings of the nodules were classified according to the American Thyroid Association 2015 guidelines (benign, very low suspicion, low suspicion, intermediate suspicion, and highly suspicion) [14]. FNA was either performed from the largest nodule or from another nodule if more suspicious. The patients usually had only one nodule aspirated since the majority of the patients did not have health insurance and could not afford the cost of multiple aspirations. Nodule size was grouped as < 1.0 cm, 1–2 cm, 2.1–4 cm, and >  4 cm. FNA reports were classified according to the Bethesda System. Patients with Bethesda IV, V, VI were referred for surgery unless the patient had other underlying diseases that caused the surgery to be high risk for the patient. Those with Bethesda III either underwent surgery or were followed up with repeat FNA. Some patients with Bethesda II or I opted for surgery either because of pressure symptoms in the neck, the large size of a goiter or hyperthyroidism. For those who underwent surgery and had a thyroid tumor, the World Health Organization (WHO) 2017 classification was used to classify thyroid tumors [15]. Thyroid cancer was classified as papillary thyroid cancer (PTC; classic, follicular, encapsulated follicular variant or papillary microcarcinoma), follicular carcinoma (FTC; minimally invasive or invasive), medullary carcinoma, anaplastic carcinoma or lymphoma, and other types of thyroid cancer were not found among our patients. Benign tumors were classified as follicular adenoma, and Hurthle-cell adenoma or borderline (premalignant) tumors were classified as follicular tumors of uncertain malignant potential, well differentiated tumors of uncertain malignant potential or noninvasive follicular neoplasms with papillary-like nuclear features.

### Statistical analysis

All statistical analyses were performed using IBM SPSS statistics (version 23). The results of the descriptive analysis of the epidemiological data were presented as frequencies, percentages and mean ± standard deviation for normally distributed data and median ± interquartile range for non-normally distributed data. The overall and sex-specific prevalence of cancerous nodules in the different age groups was reported with 95% confidence intervals (CI). Chi-square and Fisher tests were used to determine the significance of the association between the variables associated with thyroid cancer. A *P*-value of less than 0.05 was considered statistically significant.

## Results

A total of 550 patients of all age groups were included in this study (Table 1). The mean age of the patients was 38.5 (SD 12.2). Of the 550 patients (501 females and 49 males), 543 underwent FNA, and 7 underwent surgery without FNA. Surgery was ordered in 204 patients (172 had surgery, 19 refused surgery or delayed it, and 13 were lost to follow up). Among those who had surgery, 87 had benign tumors (50.6%), 76 had thyroid cancer (44.2%), and 9 had a borderline (premalignant) diagnosis (5.2%).

**Table 1:** General characteristics of the patients (n = 550)

Variable	Mean (SD)	Median (IQR)	N	%
Age (years)	38.5 (12.2)			
< 20			25	4.5
21–30			150	27.3
31–40			181	32.9
41–50			112	20.4
51–60			62	11.3
> 60			20	3.6
Sex				
Male			49	8.9
Female			501	91.1
Medications				
No medications			482	87.6
Thyroxine			47	8.5
Anti-thyroid medications			21	3.8
TSH (mIU/L)		1.3 (1.2)		
< 0.40			50	9.1
0.40–0.99			120	21.8
1.00–1.79			205	37.3
1.80–4.50			138	25.1
> 4.50			37	6.7

The number of patients who had thyroid cancer was 76 out of a total of 550, which was 13.8% of the patients who had thyroid nodules.

The patients had a median TSH of 1.3 (IQR 1.2). Of all the patients, 84.2% had a TSH level in the range of 0.4–4.5 mIU/L, 9.1% had a low TSH level (< 0.4 mIU/L) and 6.7% had a high TSH level (> 4.5 mIU/L). A total of 482 patients (87.6%) were not on any thyroid medications; 8.5% of patients had hypothyroidism and were on L-thyroxine, and 3.8% had hyperthyroidism and were on carbimazole.

The characteristics of the nodules can be seen in Table 2. The percentage of patients with a single nodule was 45.6%, and the percentage of patients with multiple nodules was 54.4%. Over half of the patients (59.3%) had a nodule size of 2.1–4 cm, and among our patients, the ultrasound reports stated low suspicion of malignancy in 55.6% of patients, intermediate suspicion of malignancy in 25.1% of patients and high suspicion of malignancy in 9.1% of patients.

**Table 2:** Characteristics of the nodules and FNA cytology according to the Bethesda system

Variable		Mean (SD)	N	%
Nodule number				
	Single		251	45.6
	Multiple		299	54.4
Nodule size (cm)		3.4 (1.4)		
	< 1 cm		1	0.2
	1.0–2.0		90	16.4
	2.1–4.0		326	59.3
	> 4.0		133	24.2
Radiology				
1	Benign		1	0.2
2	Very low suspicion		55	10.0
3	Low suspicion		306	55.6
4	Intermediate suspicion		138	25.1
5	High suspicion		50	9.1
FNA ordered				
	Yes		543	98.7
	No		7	1.3
FNA result				
Bethesda I	Nondiagnostic		18	3.3
Bethesda II	Benign		320	58.9
Bethesda III	AUS/FLUS		77	14.2
Bethesda IV	FN/SFN		81	14.9
Bethesda V	Suspicious of malignancy		21	3.9
Bethesda VI	Malignant		26	4.8

UG-FNA cytology was reported using the Bethesda system. A diagnostic category (DC) of “nondiagnostic” was noted in 18 patients (3.3%), and repeat FNA did not reveal any malignancy in these patients. DC II (benign) occurred in 320 patients (58.9%), DC III (atypia of undetermined significance and follicular lesion of undetermined significance) occurred in 77 patients (14.2%), DC IV (follicular neoplasm/suspicious for follicular neoplasm) occurred in 81 patients (14.9%), DC V (suspicious for malignancy) occurred in 21 patients (3.9%), and DC VI (malignant) occurred in 26 patients (4.8%).

AUS/FLUS: atypia of unknown significance/follicular lesion of unknown significance; FN/SFN: follicular neoplasm/suspicious for follicular neoplasm.

In Tables 3 and 4, the association of the different variables with thyroid cancer is shown. The overall prevalence of thyroid cancer was 13.8% (CI 10.9–16.7). Among the patients, 18.4% (N = 9) of males and 13.4% (N = 67) of females had thyroid cancer; this difference was not significant (*P* -value 0.334). The age group with the highest rate of cancer was 21–30 years (16.7%; CI 10.7–22.6), followed by 31–40 years (13.3%; CI 8.3–18.2). According to the number of nodules, 16.7% (N = 42) of those with single nodules had thyroid cancer, and 11.4% (N = 34) of the patients with multiple nodules had thyroid cancer; this difference was not significant (*P*-value 0.07). The TSH level was significantly related to malignancy *(P*-value 0.01). The highest rate of malignancy according to the size of the nodule was in those with 1–2 cm nodules (16.7%) followed by those with > 4 cm nodules (14.3%).

**Table 3:** Overall and sex-specific prevalence of thyroid cancer (95% CI) (*N* = 550)

Variable	Overall (*n* = 550)		Male (*n* = 49)		Female (*n* = 501)		*p*-value
	N	% (95% CI)	N	% (95% CI)	N	% (95% CI)	
Cancer (yes/no)	76/474		9/40		67/434		
All thyroid nodules		13.8 (10.9–16.7)		18.4 (7.5–29.2)		13.4 (10.4–16.4)	0.334
Age (years)							
< 20	4	16.0 (1.6–30.4)	0	0.0 (0.0–0.0)	4	18.2 (2.1–34.3)	0.420
21–30	25	16.7 (10.7–22.6)	4	36.4 (7.9–64.8	21	15.1 (9.2–21.1)	0.069
31–40	24	13.3 (8.3–18.2)	4	23.5 (3.4–43.7)	20	12.2 (7.2–17.2)	0.190
41–50	14	12.5 (6.4–18.6)	1	8.3 (0.0–24.0)	13	13.0 (21.9–40.1)	0.644
51–60	7	11.3 (3.4–19.2)	0	0.0 (0.0–0.0)	7	12.1 (3.7–20.5)	0.461
> 60	2	10.0 (0.0–23.2)	0	0.0 (0.0–0.0)	2	11.1 (0.0–25.6)	0.619

**Table 4:** Association between thyroid cancer and other variables (*n* = 550)

Variable		Cancer		No cancer		풳2	*p*-value
		N	%	N	%		
TSH (mIU/L)						13.22	0.010
	< 0.40	7	14.0	43	86.0		
	0.40–0.99	11	9.2	109	90.8		
	1.00–1.79	21	10.2	184	89.8		
	1.80–4.50	31	22.5	107	77.5		
	> 4.50	6	16.2	31	83.8		
Nodule number						3.29	0.070
	Single	42	16.7	209	83.3		
	Multiple	34	11.4	265	88.6		
Nodule size						1.04	0.792
	< 1	0	0.0	1	100.0		
	1.0–2.0	15	16.7	75	83.3		
	2.1–4.0	42	12.9	284	87.1		
	> 4.0	19	14.3	114	85.7		
Medications						3.25	0.197
	Thyroxine	9	19.1	38	80.9		
	Antithyroid	5	23.8	16	76.2		
	No medications	62	12.9	420	87.1		
Bethesda stage						160.92	< 0.001
	I	0	0.0	18	100.0		
	II	4	1.3	316	98.8		
	III	16	20.8	61	79.2		
	IV	22	27.2	59	72.8		
	V	11	52.4	10	47.6		
	VI	18	69.2	8	30.8		

When assessing the rate of cancer among the different Bethesda system categories, none of the patients in DC I had cancer. Of DC II patients, 1.3% had thyroid cancer, and in DC III, 20.8% of patients had thyroid cancer; in DC IV, V, VI, the rates of cancer were 27.2, 52.4 and 69.2%, respectively (*P*-value < 0.001).

The distribution of the different types of thyroid cancer according to age, sex and thyroid nodule size is shown in Table 5.

**Table 5:** Thyroid cancer types according to the patients’ age, sex and nodule size

Variable		PTC								FTC				Medullary cancer		Lymphoma		*p*-value
		Classic variant		Follicular variant		Encapsulated follicular variant		Micro carcinoma		Minimally invasive		Widely invasive						
		N	%	N	%	N	%	N	%	N	%	N	%	N	%	N	%	
Age (years)																		**0.726**
	**< 20**	**3**	**75.0**	**0**	**0.0**	**0**	**0.0**	**0**	**0.0**	**0**	**0.0**	**0**	**0.0**	**1**	**25.0**	**0**	**0.0**	
	**21–30**	**8**	**32.0**	**4**	**16.0**	**1**	**4.0**	**4**	**16.0**	**4**	**16.0**	**3**	**12.0**	**1**	**4.0**	**0**	**0.0**	
	**31–40**	**6**	**25.0**	**7**	**29.2**	**1**	**4.2**	**5**	**20.8**	**4**	**16.7**	**1**	**4.2**	**0**	**0.0**	**0**	**0.0**	
	**41–50**	**5**	**35.7**	**3**	**21.4**	**0**	**0.0**	**0**	**0.0**	**2**	**14.3**	**3**	**21.4**	**1**	**7.1**	**0**	**0.0**	
	**51–60**	**3**	**42.9**	**1**	**14.3**	**0**	**0.0**	**1**	**14.3**	**0**	**0.0**	**1**	**14.3**	**0**	**0.0**	**1**	**14.3**	
	**> 60**	**2**	**100.0**	**0**	**0.0**	**0**	**0.0**	**0**	**0.0**	**0**	**0.0**	**0**	**0.0**	**0**	**0.0**	**0**	**0.0**	
Sex																		**0.132**
	**Male**	**1**	**11.1**	**1**	**11.1**	**1**	**11.1**	**2**	**22.2**	**1**	**11.1**	**2**	**22.2**	**1**	**11.1**	**0**	**0.0**	
	**Female**	**26**	**38.8**	**14**	**20.9**	**1**	**1.5**	**8**	**11.9**	**9**	**13.4**	**6**	**9.0**	**2**	**3.0**	**1**	**1.5**	
Nodule size (cm)																		**0.372**
	**< 1 cm**	**0**	**0.0**	**0**	**0.0**	**0**	**0.0**	**0**	**0.0**	**0**	**0.0**	**0**	**0.0**	**0**	**0.0**	**0**	**0.0**	
	**1.0–2.0**	**7**	**46.7**	**2**	**13.3**	**0**	**0.0**	**3**	**20.0**	**3**	**20.0**	**0**	**0.0**	**0**	**0.0**	**0**	**0.0**	
	**2.1–4.0**	**17**	**40.5**	**9**	**21.4**	**2**	**4.8**	**3**	**7.1**	**3**	**7.1**	**5**	**11.9**	**2**	**4.8**	**1**	**2.4**	
	**> 4.0**	**3**	**15.8**	**4**	**21.1**	**0**	**0.0**	**4**	**21.1**	**4**	**21.1**	**3**	**15.8**	**1**	**5.3**	**0**	**0.0**	

In the younger patients (age < 20), the PTC classic variant made up 75% of the patients, and medullary cancer was present in the remaining 25% of patients. Widely invasive follicular cancer occurred in 21.4% of thyroid cancer cases in patients aged 41–50 years. In patients aged 51–60 years, the classic variant of PTC occurred in 42.9% of cases, and in patients > 60 years old, all the cases were the classic variant of PTC.

Males were found to have a higher rate of papillary microcarcinomas, widely invasive FTC and medullary cancer than females.

The distribution of the different types of cancer in relation to the size of the nodules on ultrasound before FNA. None of the patients had a nodule < 1 cm in size on ultrasound because these patients were not sent for FNA. The highest percentages of PTC microcarcinomas were found in the 1–2 cm group and in the > 4 cm group. Additionally, in the larger nodules (> 4 cm), we found a higher rate of FTC (21.1% minimally invasive and 15.8% widely invasive). This rate was higher than the rate of follicular cancer in smaller nodules.

The ultrasound report on thyroid nodules and the number of patients with malignancy can be seen in Table 6. Among the patients with benign and very low suspicion of malignancy reports on ultrasound, none of them had thyroid cancer. Among the patients with an ultrasound report of low suspicion for malignancy, 36.5% had thyroid cancer; among the patients with intermediate suspicion for malignancy, 42.7% had thyroid cancer; and among the patients who had a high suspicion for malignancy, 70% had cancer, and 3.3% had a premalignant diagnosis.

**Table 6:** Sonographic report on thyroid nodules and rate of thyroid cancer (*n* = 172)

Radiology	Benign		Malignant		Borderline	
	N	%	N	%	N	%
Benign	0	0.0	0	0.0	0	0.0
Very low suspicion	4	100.0	0	0.0	0	0.0
Low suspicion	38	60.3	23	36.5	2	3.2
Intermediate suspicion	37	49.3	32	42.7	6	8.0
High suspicion	8	26.7	21	70.0	1	3.3

The number of patients who underwent surgery and the biopsy results after surgery are shown in Table 7. Surgery was ordered in 204 patients (37.1%); the majority of the patients had a total thyroidectomy, and out of these, 172 patients had surgery (84.3%). Of the remaining patients, 19 (9.3%) did not undergo surgery, and 13 (6.4%) were lost to follow-up. Among those who had surgery, 87 (50.6%) had a benign biopsy result, 76 (44.2%) had a malignant result, and 9 (5.2%) had a borderline result (premalignant diagnosis).

**Table 7:** The number of patients who underwent surgery and the biopsy results after surgery (*N* = 172)

Variable	N	%
Surgery ordered		
Yes	204	37.1
No	346	62.9
Surgery done		
Yes	172	84.3
No	19	9.3
Lost to follow up	13	6.4
Biopsy result		
Benign	87	50.6
Malignant	76	44.2
Borderline (premalignant)	9	5.2
Benign tumors		
Follicular adenoma	7	77.8
Hurthle cell adenoma	2	22.2
Borderline		
NIFTP	2	22.2
Follicular tumor of unknown malignant potential	3	33.3
Well differentiated tumor of unknown malignant potential	4	44.4
Malignant type		
PTC classic variant	27	35.5
PTC follicular variant	15	19.7
PTC encapsulated follicular variant	2	2.6
PTC microcarcinoma	10	13.2
FTC minimally invasive	10	13.2
FTC widely invasive	8	10.5
Medullary cancer	3	3.9
Lymphoma	1	1.3

The patients who had borderline diagnoses (*N* = 9) had well-differentiated tumors of unknown malignant potential in 4 of the cases, follicular tumors of unknown malignant potential in 3 cases and noninvasive follicular neoplasms with papillary-like nuclear features in 2 cases.

Papillary thyroid cancer (PTC) was the most common thyroid cancer, occurring in 71% of patients. The classic variant was the most common type, found in 35.5% of the cancer patients. Follicular cancer occurred in 23.7% of the patients, medullary cancer occurred in 3.9% of patients, and lymphoma occurred in 1.3% of the patients. There were no cases of anaplastic carcinoma among our patients.

NIFTP: noninvasive follicular neoplasm with papillary-like nuclear features; PTC: papillary thyroid cancer; FTC: follicular thyroid cancer.

Of patients with hyperthyroidism (*N* = 21), 5 were found to have cancer. Of the patients who were lost to follow-up after having an UG-FNA (*N* = 13), 8 had a classification of DC V or VI (Additional file 1).

## Discussion

The prevalence of thyroid cancer is increasing worldwide. This is the first study of the prevalence of thyroid cancer among Yemeni patients with thyroid nodules during Oct. 2016 to Oct 2019 in a specialized endocrinology clinic and in an endocrinology clinic in a tertiary hospital in Sana’a, Yemen; all of the patients were seen by the same endocrinologist. A total of 550 patients who had UG-FNA and/or surgery were seen. A high prevalence of thyroid cancer was seen in our patients with thyroid nodules (13.4% of the females and 18.4% of the males), and there was no significant difference in the rate of cancer among those with single nodules and multiple nodules (P value = 0.07); this is similar to what was reported by Frates et al. [16], and the highest rate of cancer was among those with nodules 1–2 cm in size; this is similar to a study that showed the highest malignancy rate among nodules < 2 cm in size [17]. The overall prevalence of cancer among patients with thyroid nodules was 13.8%, which is higher than that in neighboring countries, which is reported to be approximately 9–10% [8–10]. TSH level was significantly associated with thyroid cancer (*P* -value 0.01), which is consistent with studies that showed an increased risk of thyroid cancer with an increase in TSH level [12, 18].

Among the male patients, FTC accounted for 33% of the cancer cases, whereas in females, FTC accounted for 22.4% of the cases. This is different than a study in the USA that showed the FTC rate among females to be twice the rate in males [19]. In our patients, FTC was the most prevalent in patients aged 41–50 years, which is younger than the age reported in a study in the USA that showed that FTC rates were highest in older age groups in both females and males [19].

Patients with nodules > 4 cm in size accounted for 36.9% of all FTC cases, whereas no patients with tumors < 2 cm had FTC in our study. A low rate of follicular cancer in patients with smaller nodules has also been shown in other studies [20].

The Bethesda System is used for reporting thyroid cytopathology [4]. In our patients, the rate of malignancy among the different categories was slightly different than that reported in other studies among those in DCI (nondiagnostic) after repeating the FNA; the result was benign in all cases, with one remaining nondiagnostic the 2nd time and with none of the cases being malignant; in another study, the rate of cancer in nondiagnostic FNAs was 5% [21]. Of DC II (benign) nodules, 1.3% were found to be malignant, which is similar to that reported by Cibas al [4]. The rate of thyroid cancer among those with DCIII (AUS/FLUS) nodules was 20.8%, which is higher than the rates in other studies that reported a 5–15% risk of cancer [4, 5]; the rate of cancer among DCIV (FN/SFN) nodules was 27.2% in our patients, which is within the expected rate of 15–30% [4]. Among patients with DCV and DCVI nodules, the rate of cancer in our patients was lower than expected (52.4 and 69.2%, respectively), which may be due to the inclusion of FNA reports from different pathology labs that may not have much experience with thyroid cytology.

The ultrasounds performed in the endocrinology clinic were reviewed by the radiologist in the center to ensure that the staging was correct. For patients with ultrasounds performed in the hospital or patients who came from other centers with an ultrasound report, we depended on the report for the ultrasound staging [14]. For those with a highly suspicious sonographic finding, 70% of our patients were found to have cancer, which is close to the estimated risk of malignancy (> 70–90%) among patients with similar sonographic findings [14].

A small number of the patients in our study had hyperthyroidism (*N* = 21), of whom 23.8% (*N* = 5) had thyroid cancer. In a study performed on a larger number of patients with Graves’ disease and thyroid nodules, the rate of cancer was 13% [22], so it should be kept in mind that these nodules should be assessed the same way regardless of hyperthyroidism if a nodule is suspicious on ultrasound.

Analysis of the distribution of the different types of cancer in our patients showed that the prevalence of papillary cancer was slightly less than that reported elsewhere. In our patients, 71% were found to have papillary cancer, and 23.7% were found to have follicular cancer. This is different than that the results reported in Yemen in 2004, where the rate of papillary cancer among patients undergoing surgery made up 93.8% of the cases of thyroid cancer, and only 4.1% had follicular cancer [23]. In other countries, PTC accounts for 85–90% of all thyroid cancers, with the highest rate in Korea at 95% [10, 11, 13, 24]. The increased rate of PTC worldwide is multifactorial, but iodization of salt is considered one of the causes [1]. Salt iodization was introduced in Yemen in 1995 and was, at that time, supposed to cover 22–60% of the households. Whether this is still the case is unknown; this may be a factor causing an increase in the percentage of patients with FTC. The war started in Yemen in 2015 with increased bombing in many areas of the country. This could be a source of environmental carcinogens causing an increase in the rate of thyroid cancer, including an increase in the risk of FTC; this has to be further studied, but environmental pollutants have been linked to an increase in thyroid cancer [25, 26].

The limitations of our study are that all these patients were seen by one endocrinologist in 2 different centers. Due to the small number of endocrinologists in the country, there may be a referral bias. Some of the patients (4.2%) were referred after having an UG-FNA or surgery in another center. Some of the patients did not return for follow-up after surgery was ordered, and we were not able to contact them. Among these, 8 patients had Bethesda classifications V and VI, which may indicate that the prevalence of cancer is even higher than calculated, but this cannot be confirmed.

## Conclusion

Our study showed that the prevalence of thyroid cancer in Yemen is higher than that in other middle eastern countries. Further studies have to be done to confirm this and to study the possible risk factors; there are no previous studies in Yemen to see if the rate of thyroid cancer is increasing. A stepwise approach to managing patients with thyroid nodules is important for decreasing the number of unnecessary surgeries. Due to the small number of endocrinologists in Yemen, the majority of patients with thyroid nodules are treated by internists and surgeons with a high rate of surgeries in patients with thyroid nodules that are benign without FNAs prior to surgery. In our study, ~ 50% of the patients who had surgery had a malignant or premalignant biopsy result. Educational programs are needed to educate physicians, especially in rural areas, on the need to approach patients with thyroid nodules with thyroid ultrasound and US-FNA when indicated. Additionally, there is a general fear among Yemeni patients on having UG-FNAs, and it is believed among the general population that having an FNA will spread the disease if it is malignant. This is usually overcome in most patients if the physician explains to the patient the benefits of having an FNA over going directly for surgery.

## Supplementary information

**Additional file 1.** Thyoid nodule study.

## Data Availability

All data are included in this published article.

## Abbreviations

UG-FNA: Ultrasound-guided fine needle aspiration
FNA: Fine needle aspiration
TSH: Thyroid stimulating hormone
DC I-VI: Diagnostic category of Bethesda classification I-VI
NIFTP: Noninvasive follicular neoplasm with papillary-like nuclear features
AUS/FLUS: Atypia of unknown significance/follicular lesion of unknown significance
FN/SFN: Follicular neoplasm/suspicious for follicular neoplasm
PTC: Papillary thyroid cancer
FTC: Follicular thyroid cancer

## References

[1] Dong W, Zhang H, Zhang P (2013). The changing incidence of thyroid carcinoma inn Shenyang, China before and after universal salt iodization. Med Sci Monit.

[2] Gharib H, Papini E, Paschke R (2008). Thyroid nodules: a review of current guidelines, practices, and prospects. Eur J Endocrinol.

[3] Muratli A, Erdogan N, Sevim S (2014). Diagnostic efficacy and importance of fine- needle aspiration cytology of thyroid nodules. J Cytol.

[4] Cibas ES, Ali SZ (2009). NCI thyroid FNA state of the science conference. The Bethesda system for reporting thyroid cytopathology. Am J Clin Pathol.

[5] Gao LY, Ying W, Yu-Xin J (2017). Ultrasoud is helpful to differentiate Bethesda class III thyroid nodules a PRISMA-compliant systematic review and meta-analysis. Medicine.

[6] Ferlay J, Soerjomataram I, Dikshit R (2015). Cancer incidence and mortality worldwide: sources, methods and major patterns in GLOBOCAN 2012. Int J Cancer.

[7] Oh CM, Kong HY, Kim E et al. National Epidemiologic Survey of Thyroid cancer (NEST) in Korea. epiH 2018;40: e2018052. 10.4178/epih.e2018052 PMC6335496 30376709

[8] Abdullah N, Hajeer M, Abudalu L (2018). Correlation study of thyroid nodule cytopathology and histopathology at two institutions in Jordan. Cytojournal.

[9] Saeed MI, Hassan AA, Butt ME (2018). Pattern of thyroid lesions in Western region of Saudi Arabia: a retrospective analysis and literature review. J Clin Med Res.

[10] Al Dawish MA, Robert AA, Aljuboury M (2017). Bethesda system for reporting thyroid cytopathology: a three-year study at a tertiary care referral center in Saudi Arabia. World J Clin Oncol.

[11] Alseddeeqi E, Baharoon R, Mohamed R (2018). Thyroid malignancy among patients with thyroid nodules in the United Arab Emirates: a five-year retrospective tertiary Centre analysis. Thyroid Res.

[12] Khan MA, Malik N, Khan KH (2017). Association of preoperative serum thyroid-stimulating hormone levels with thyroid cancer in patients with nodular thyroid disease. World J nucl Med.

[13] Shi X, Liu R, Basolo F (2016). Differential Clinicopathological risk and prognosis of major papillary thyroid cancer variants. J Clin Endocrinol Metab.

[14] Haugen BR, Alexander EK, Bible KC (2016). 2015 American Thyroid Association management guidelines for adult patients with thyroid nodules and differentiated thyroid Cancer. Thyroid.

[15] Bychkov A. World Health Organization (WHO) classification. Pathology Outlines.com website. http://www.pathologyoutlines.com/topic/thyroidwho.html. Accessed December 21^st^, 2019.

[16] Frates MC, Benson CB, Doubilet PM (2006). Prevalence and distribution of carcinoma in patients with solitary and multiple thyroid nodules on sonography. J Clin Endocrinol Metab.

[17] Cavallo A, Johnson DN, White MG (2017). Thyroid nodule size at ultrasound as a predictor of malignancy and final pathologic size. Thyroid.

[18] Boelaert K, Horacek J, Holder RL (2006). Serum thyrotropin concentration as a novel predictor of malignancy in thyroid nodules investigated by fine-needle aspiration. J Clin Endocrinol Metab.

[19] Aschefrook-kilfoy B, Grogan RH, Ward MH (2013). Follicular thyroid Cancer incidence patterns in the United States, 1980-2009. Thyroid.

[20] Podda M, Saba A, Porru F (2015). Follicular thyroid carcinoma: differences in clinical relevance between minimally invasive and widely invasive tumors. World Journal of Surgical Oncology.

[21] Alexander EK, Heering JP, Benson CB (2002). Assessment of nondiagnostic ultrasound –guided fine needle aspirations of thyroid nodules. J Clin Endocrinol Metab.

[22] Tam AA, Kaya C, Balkan F (2014). Thyroid nodules and thyroid cancer in graves’ disease. Arq Bras Endocrinol Metab.

[23] Abdulmughni YA, Al-Hureibi MA, Al-Hureibi KA (2004). Thyroid cancer in Yemen. Saudi Med J.

[24] Mimi K, Pack HJ, Min HS (2017). The use of Bethesda system for reporting thyroid cytopathology in Korea: a nationwide multicenter survey by the Korean society of endocrine pathologists. Journal of Pathology and Translational Medicine.

[25] Pellegriti G, Frasca F, Regalbuto C, et al. Worldwide increasing incidence of thyroid cancer: update on epidemiology and risk factors. Journal of Cancer Epidemiology. 2013;965212. 10.1155/2013/965212 PMC3664492 23737785

[26] Tuminello S, van Gerwin MAG, Genden E (2019). Increased incidence of thyroid cancer among world trade center first responders: a descriptive epidemiological assessment. Int J Environ Res Public Health.

